# Psychological well-being among Canadian chiropractors: a cross-sectional questionnaire-based study

**DOI:** 10.1186/s12998-025-00616-w

**Published:** 2025-11-28

**Authors:** Gregory Neil Kawchuk, Silvano Mior, Crystal Draper, Mingyang Li, W. Dominika Wranik

**Affiliations:** 1 Department of Physical Therapy, Faculty of Rehabilitation Medicine, University of Alberta, Edmonton, Canada; 2 Department Research and Innovation, Canadian Memorial Chiropractic College, Toronto, ON Canada; 3 Institute for Disability and Rehabilitation Research, Ontario Tech University, Oshawa, Canada; 4 Canadian Chiropractic Association, Toronto, Canada; 5 Department of Public and International Affairs, Faculty of Management, Dalhousie University, Halifax, Canada; 6 Department of Community Health and Epidemiology, Faculty of Medicine, Dalhousie University, Halifax, Canada

**Keywords:** Chiropractic, Mental health, Depression, Anxiety, Burnout, Suicidal ideation, Health occupations, Psychological well-being

## Abstract

**Background:**

Chiropractors are trained to help others, but do chiropractors need help themselves? While many health professions have been surveyed regarding their mental health and wellness, no such comprehensive survey has been conducted in the chiropractic profession. These data are important to improve the health of individual chiropractors, designing effective interventions and creating a strategic workforce plan.

**Methods:**

We used a cross-sectional design to survey Canadian chiropractors and students between 02–2023 and 04–2023. We adapted the Canadian Medical Association’s questionnaire with permission. The questionnaire was divided into 5 domains; we report results from the psychological domain. Descriptive statistics were tabulated and inferential statistics conducted on each psychological test stratified by gender, age, years and type of practice, and geographic location. From these data, most-at-risk and least-at-risk profiles were constructed.

**Results:**

Anonymous data were obtained from 2109 respondents (26% response rate). The majority identified as male, white and without disability. Respondents identifying as practicing chiropractors and students represented 92.6% and 4.5% of the sample, respectively. From the mental health continuum, 57.8% of Canadian chiropractors were considered to be flourishing and only 5.3% languishing. Chiropractors reported experiencing substantial levels of mental health issues including burnout (33.2%), anxiety (22.0%), and lifetime depressed mood (36.8%). Most notably, 26.7% of responding chiropractors reported having lifetime suicidal ideation. Respondents identifying as having a disability (20%) or being students reported more unfavourable scores across all psychological measures. The most at-risk profile for a Canadian chiropractor is a young female practitioner (under 31) with less than 5 years of experience. In contrast, the least at-risk profile is an older male (over 60) with 31 or more years of practice experience.

**Conclusion:**

This is the first comprehensive evaluation of mental health and wellness in the Canadian chiropractic profession. The results demonstrate that, like other professions, there are mental health and wellness challenges among Canadian chiropractors. These data have value in designing preventative and intervention strategies for those most at-risk. Currently, this survey is being conducted in other countries to understand the mental health of the chiropractic profession in different settings. Future work will analyze the remaining domains.

**Supplementary Information:**

The online version contains supplementary material available at 10.1186/s12998-025-00616-w.

## Introduction

*Medice, cura te ipsum*. 

Healthcare professionals commonly experience mental health disorders such as stress, burnout, and addiction, often at greater rates than the general public. For example, in 2017, 34% of Canadian physicians and 36% of nurses [1] were estimated to have symptoms of depression compared to approximately 14% of the general population [2]. Such mental health disorders have consequences and contribute to increased work attrition, low job satisfaction, and decreased productivity [3]. The consequences related to the mental health of health professions and their members are well known, and public health disasters such as pandemics increase emotional and psychological distress and burnout [4], bring into sharp focus that the public’s healthcare and well-being is dependent on the well-being of its providers.

The mental health status of mainstream health professions such as physicians and nurses has been studied before, during and post-pandemic [5–7]. Specifically, the Canadian Medical Association (CMA) conducted its first comprehensive survey of mental health and wellness in 2017 [8] and showed physicians are at a high risk of developing symptoms of burnout, depression and other psychological distress. The survey was repeated in 2021 during the COVID-19 pandemic [9] and found a further decline in physician mental health. Similar findings have been reported in nursing. The American Nurses Foundation has conducted four mental health and wellness surveys, the most recent in May of 2023 [10]. Their findings show that 56% of respondents experience burnout, 64% reported significant job-related stress, and 39% intend to leave their current position within the next six months.

Most investigations in this area have studied the medical and nursing professions, but to date, little is known about the well-being and mental health of other healthcare professions, particularly the chiropractic profession. The chiropractic profession has been regulated in Canada, beginning in Alberta, since 1923 [11]. Chiropractors are the most utilized complementary and alternative medicine (CAM) primary care providers and the second most utilized health profession for the management of chronic back pain [12]. Yet, few efforts have been made to understand the mental health and wellness of chiropractors in Canada or globally [13]. Although there have been several reports that have characterized occupational demographics of the profession, e.g., numbers serving geographic areas, average income [14–18], reports describing work-related injuries [17, 19] and recently, a systematic review of low back pain in chiropractors [20], there are few reports that describe the well-being or mental health of chiropractors.

One such report is a 2024 scoping review examining studies related to burnout amongst chiropractic practitioners, faculty and students [13]. The review included 10 papers, two narrative reviews, one survey of faculty, three of students and four of licensed chiropractors, with the largest sample size being 1162 [21]. Themes consistent with other health professions were identified as impacting burnout, such as issues related to scope of practice, reimbursements, administrative duties and negative public opinion of the profession. None of the included studies were based on a national-level survey, none were Canadian, and aside from 6 of the 10 studies assessing burnout using the Maslach Burnout Inventory, none included important validated measures of mental health, such as depression, anxiety, and suicidal ideation [13], each known to impact healthcare professionals [22].

Therefore, the aim of our study was to describe the state of well-being and mental health among Canadian chiropractors and students, and to identify sub-groups who are at particular risk of adverse outcomes. This knowledge is important for three reasons. First, it allows for the design of meaningful interventions for its students and professionals. Second, at a system level, it can inform key stakeholders, including academic institutions, professional associations and regulatory bodies, to undertake programs to manage the profession’s current and future workforce. Third, it is critical to ensure high quality and continuity of care for the patients they serve.

## Methods

### Study design

We conducted a cross-sectional descriptive study of the well-being and mental health of chiropractors in Canada. This design lends itself to the independent collection of comparative data in other locations and/or of other professions, as well as follow-up data at future points in time. Our study was adapted from the 2017 [8] and 2021 [9] surveys conducted by the Canadian Medical Association (CMA) and used with their permission (see Additional File 1 for details). Adaptations made to the questionnaire ensured its relevance to the chiropractic profession and aligned with previous studies involving the Canadian Chiropractic Association (CCA) [23]. This adaptation was necessary as specific questions in the CMA survey would not be relevant to non-physician healthcare professionals. Some adaptations were made so that the data collected would align with prior data collected by the Canadian Chiropractic Association (CCA) (e.g., age categories, ethnicity, Indigeneity, disability/impairment/long-term condition, caretaker status, chiropractic college attended and payment source(s)).

Ethical approvals were granted by the Human Research and Ethics Board of the University of Alberta (Pro00124711), Dalhousie University (REB #2025–8078), and the Canadian Memorial Chiropractic College (REB Project no. 2211 × 01).

### Questionnaire development

Building on the CMA questionnaire, we adapted wording and piloted the CCA questionnaire during the National Convention and Tradeshow in Montreal in October 2022. Feedback from participants (n = 349) was used to further refine the survey. The final version consisted of 65 questions containing 175 items, organized into five domains: demographics and occupational characteristics, psychological well-being, impact of COVID-19, behavioural factors, and social supports. The questionnaire was made available in both English and French languages and was disseminated to chiropractors and chiropractic students through the CCA and the Student Canadian Chiropractic Association (SCCA), respectively. To identify questions aimed at chiropractors and/or chiropractic students, we used clarifying language in the question prompt and provided a “prefer not to answer” response. This article focuses on the psychological well-being and demographics reported by participant chiropractors and students.

### Data collection

All members of the CCA, who identified as either English (n = 6311) or French (n = 824) were invited to participate in the study via an email message with a link to the questionnaire. In addition, students from the Canadian Memorial Chiropractic College (CMCC) (n = 792) and the Université du Québec à Trois-Rivières (n = 220) were invited to participate via email distribution of the survey invitation. The survey was administered using the REDCap platform and was open from 02–2023 to 04–2023. A communication and distribution plan devised and implemented by the CCA, based on a previously conducted survey [23], was created to promote the study before its launch, during the open response period and just before the survey closed. The open link format of survey distribution ensured respondent privacy in that no personal identifiers were used to send the survey link or in the collection of data. None of the questions had mandatory response fields, and warnings were embedded in the survey so respondents could skip sensitive sections if desired (e.g., questions on suicidal ideation). At various points in the survey, the respondents were made aware of available mental health resources that could be accessed if they experienced psychological or emotional discomfort and felt help was desired. If so desired, they were directed to the site: Together All and/or Wellness Together Canada: https://www.wellnesstogether.ca/en-CA.

### Measures

The core outcome in this study is psychological well-being, and was assessed by measuring five concepts, four of which relied on validated published instruments. Each is described in more detail below.

#### Mental health and well-being

This concept refers to a ‘syndrome of symptoms of positive feelings and positive functioning in life’ (23, p. 208) and was measured using the Mental Health Continuum Short Form (MHC-SF) that consists of 14 items assessing well-being [24]. The MHC-SF was tested and validated in the Canadian context [25]. The MHC is comprised of three dimensions, including emotional, social, and psychological well-being, each with a six-point scale from “never” to “every day” [26], allowing for a scoring of respondents along each dimension, as well as overall mental health. Keyes [26] proposed a categorical scoring, where respondents scoring in the top tertile on at least one (of three) of the emotional well-being questions, and at least six (of eleven) of the psychological and social well-being questions could be classified as flourishing, those scoring in the bottom tertiles as languishing, and those scoring in the middle as moderate mental health. The MHC-SF has also been scored continuously from 14 to 84 for the full scale, 3 to 18 for the emotional subscale, 5 to 30 for the psychological subscale, and 6 to 36 for the social subscale [27]. We used and reported both scoring approaches in our study.

#### Anxiety

This concept was measured using the Generalized Anxiety Disorder 7-Item (GAD-7) instrument [28]. The GAD-7 was designed for clinical settings to screen patients for anxiety disorder and measure its severity. It consists of 7 questions related to symptoms over the past two weeks, including feeling nervous, inability to stop worrying, and physical symptoms like restlessness. Respondents identify the frequency of experiencing the symptoms on a four-point scale from “not at all” to “nearly every day” [28]. The scores range from 0 to 21, with a higher score indicating higher anxiety levels. In line with Spritzer et al., scores are interpreted as minimal (0–4), mild (5–9), moderate (10–14), and severe (15–21) [28].

#### Burnout

The experience of burnout was measured using the two-item version [29] of the original 22-item Maslach Burnout Inventory [30]. This instrument measures burnout along two dimensions: emotional exhaustion and depersonalization, each along a seven-point Likert scale ranging from “never” to “every day”. The two-item version [29] asks for the frequency of experiencing the feeling of burnout from one’s work or training environment and having become more callous towards people since taking the job or starting training. In line with West et al., scores for individual items are interpreted as high (once a week or more) and low otherwise [29].

#### Depression

The presence of depressive symptoms was measured using the two-item version (PHQ-2) [31] of the original nine-item (PHQ-9) Patient Health Questionnaire [32]. The PHQ-2 was designed as an efficient screening and triage tool for primary care clinical settings. The PHQ-2 was used in the questionnaire to inquire about the presence or absence of anhedonia (PHQ-2.1) and depressed mood (PHQ-2.2) lasting two weeks or more anytime during the respondent’s lifetime. Results are reported as binary (yes/no) variables, focusing on the percentage reporting the presence of anhedonia or depressed mood.

#### Suicidal ideation

Evaluation of suicidal ideation was assessed with two bespoke questions. The first, “Have you had thoughts of suicide?” allowed for 6 responses: Yes, before my chiropractic training/education; Yes, during my chiropractic training/education; Yes, during additional training/education after chiropractic college; Yes, during chiropractic practice; No, I have never had thoughts of suicide; I prefer not to answer. The second question, “Have you had thoughts of suicide in the last 12 months?” provided 3 responses: Yes, No, and I prefer not to answer.

#### Demographics and practice characteristics

We collected data regarding gender, age, ethnicity, racial identity, indigenous identity, career stage, primary area of practice, years in practice, practice location, practice setting, modes of financial reimbursement, personal disability and caregiving duties. Response options were kept consistent with those in the CMA’s survey or aligned with variable levels used previously in the CCA surveys [23].

### Data analysis

Data cleaning and analysis were conducted via custom code written in R using RStudio [33]. For the latent dependent variables, including mental health, burnout and anxiety, the response categories were collapsed and scorings were classified in accordance with the respective original validation studies [26, 28, 30, 31]. The frequency and percentage frequency of respondents experiencing flourishing/ moderate/ languishing mental health, high/low burnout, minimal/mild/moderate/severe anxiety are reported by the demographic categories along gender, age, area of practice, years in practice, community size, practice format/setting, disability, caregiver status, and primary payment sources. To assess whether differences in response categories for mental health, burnout, and anxiety between types of respondents were significant, chi-squared tests were performed. In addition to comparing these scores in categories, their respective continuous scores were summarized as means and standard deviations by collapsed respondent characteristics. Group differences were evaluated using ANOVA when assumptions of normality and homogeneity of variance were satisfied, or non-parametric tests (Wilcoxon rank-sum or Kruskal–Wallis tests) when assumptions were violated. The presence of depression and suicidal ideation were summarized as counts and percentages by collapsed respondent characteristics, and group comparisons were conducted using chi-square tests when assumptions were met, or Fisher’s exact tests if otherwise. (See Additional File 2 for further details.)

## Results

### Respondent characteristics

A total of 2109 usable responses were collected electronically, yielding an estimated response rate of 26%. Complete respondent characteristics are reported in Table 1. For later analysis, some response categories were collapsed to avoid small subgroups and potential identifiability, and the unknown categories were omitted from analysis (see Additional File 2). The majority of respondents self-identified as a man (50.7%), white (83.8%), without disability (80.2%), and currently practicing chiropractic (92.6%) with no identified sub-specialty (85.2%). Student usable responses accounted for only 4.5% of the total sample, or an 8% response rate amongst students sampled.

**Table 1:** Respondent Characteristics* (n = 2109)

Characteristics	Category	n (%)
Gender (n = 1755)	Man	889 (50.7)
	Woman	859 (48.9)
	Other (please specify)	4 (0.2)
	Trans man	1 (< 0.1)
	Trans woman	1 (< 0.1)
	Gender-fluid or non-binary	1 (< 0.1)
	Unknown	354
Age (n = 1769)	< 31	331 (18.7)
	31–40	376 (21.3)
	41–50	413 (23.3)
	51–60	375 (21.2)
	> 60	274 (15.5)
	Unknown	340
Ethnicity (n = 1767)	White	1480 (83.8)
	Chinese	61 (3.5)
	South Asian	88 (5.0)
	West Asian	13 (0.7)
	Arab	19 (1.1)
	Japanese	11 (0.6)
	Black	14 (0.8)
	Korean	5 (0.3)
	Filipino	13 (0.7)
	Southeast Asian	9 (0.5)
	Latin American	8 (0.5)
	Mixed Race	52 (2.9)
	Another visible minority group	15 (0.8)
	Prefer not to answer	38 (2.2)
	Unknown	342
Disability (multiple roles possible, n = 1733)	No disability	1390 (80.2)
	Hearing	36 (2.1)
	Speech	4 (0.2)
	Physical or mobility	43 (2.5)
	Mental health condition	94 (5.4)
	Neurodevelopmental disorder	42 (2.4)
	Chronic or long-term condition	98 (5.7)
	Other (please specify)	46 (2.7)
	Prefer not to say	47 (2.7)
	Unknown	376
Caregiver status (multiple roles possible, n = 1765)	Children under 18	676 (38.3)
	Family member (adult)	110 (6.2)
	Not a caregiver	1016 (57.6)
	Unknown	344
Career stage (n = 1785)	Currently practicing chiropractic	1653 (92.6)
	Chiropractic student	81 (4.5)
	Graduate school student	1 (< 0.1)
	Clinical resident	4 (0.2)
	On leave from practice	13 (0.7)
	Paused practice, but not retired	28 (1.6)
	Retired from practice	5 (0.3)
	Unknown	324
Area of practice (n = 1664)	General chiropractic	1418 (85.2)
	Sports chiropractic	81 (4.9)
	Rehabilitation chiropractic	72 (4.3)
	Pregnancy chiropractic	15 (0.9)
	Paediatric chiropractic	14 (0.8)
	Diagnostic radiology	1 (< 0.1)
	Administrative	9 (0.5)
	Research	3 (0.2)
	Other (please specify)	34 (2.0)
	Not applicable	12 (0.7)
	Prefer not to answer	5 (0.3)
	Unknown	445
Years in practice (n = 1664)	5 or less	369 (22.2)
	6–10 years	199 (12.0)
	11–15 years	182 (10.9)
	16–10 years	178 (10.7)
	21–25 years	253 (15.2)
	26–30 years	190 (11.4)
	31 years or more	293 (17.6)
	Unknown	445
Community size / geographic location (n = 1666)	Urban or suburban	1207 (72.4)
	Small town or rural	424 (25.5)
	Geographically isolated, remote	10 (0.6))
	Majority of practice is telehealth	4 (0.2)
	Cannot identify a primary geographic location	16 (1.0)
	Prefer not to answer	5 (0.3)
	Unknown	443
Practice format / setting (n = 1666)	Community—interdisciplinary	593 (35.6)
	Community—interdisciplinary medical	41 (2.5)
	Community—solo practice (chiropractor only)	526 (31.6)
	Community—solo/multi dc practice	45 (2.7)
	Community—interdisciplinary rehab	445 (26.7)
	Hospital outpatient	3 (0.2)
	Prefer not to answer	13 (0.8)
	Unknown	443
Primary payment source (n = 1662)	Direct out-of-pocket payment	745 (44.8)
	Extended health insurance (direct billing)	729 (43.9)
	Extended health insurance (patient reimbursement)	128 (7.7)
	MVA insurance	26 (1.6)
	Workers’ Compensation Board or equivalent	6 (0.4)
	Provincial/ Territorial public health insurance plan	2 (0.1)
	Prefer not to answer	25 (1.5)
	Unknown	447

^*^This table presents the demographic and professional characteristics of survey respondents, including gender, age, ethnicity, disability status, caregiver roles, career stage, area and format of practice, years in practice, geographic location, and primary payment source. Frequencies and percentages are reported for each category. Unknown or missing responses are noted where applicable.

Respondents were relatively evenly distributed between age groups and years in practice, worked primarily in urban or suburban locations (72.4%), in interdisciplinary (35.6%) or chiropractic-only (31.6%) practice. Most received payments through patients out-of-pocket (44.8%) or direct-billed insurance payments (43.9%) (Table 1).

### Well-being and mental health outcomes

Our general results suggest that participating chiropractors reported being in relatively good well-being and mental health, with important caveats in terms of distribution and differences between sub-groups. Results of sub-group comparisons are presented in Tables 2, 3, 4 and 5. Table 2 reports mean scores and standard deviations and sub-group comparisons for the Mental Health Continuum. Table 3 provides mean scores, standard deviations, and sub-group comparisons for Generalized Anxiety Disorder, and Burnout outcomes. Table 4 presents the frequency distributions and subgroup comparisons for the Mental Health Continuum categories—flourishing, moderate, and languishing mental health, as well as for the high and low categories of social, emotional, and psychological well-being. Table 5 includes frequency distributions and sub-group comparisons for high and low risk of burnout, emotional exhaustion, depersonalization, and the four categories of Generalized Anxiety Disorder. Table 6 reports frequency distributions and sub-group comparisons for the presence or absence of depressive symptoms and suicidal ideation.

**Table 2:** Mental health continuum-short form scale and subscale scores*

Characteristics	MHC-SF full scale (range 14–84)		MHC-SF emotional subscale (range 3–18)		MHC-SF psychological subscale (range 5–30)			MHC-SF social subscale (range 6–36)	
	n	Mean ± SD	n	Mean ± SD	n	Mean ± SD	n		Mean ± SD
All usable responses	922	61.39 ± 13.60	982	14.39 ± 3.25	969	23.28 ± 5.16	946		23.65 ± 6.67
*Gender*									
Man	454	62.00 ± 13.98	489	14.52 ± 3.32	478	23.53 ± 5.31	467		23.83 ± 6.82
Woman	459	60.80 ± 13.18	481	14.25 ± 3.14	479	23.05 ± 4.94	470		23.47 ± 6.54
Another gender	2	63.50 ± 4.95	3	16.00 ± 1.73	3	24.67 ± 6.11	2		25.00 ± 1.41
*p*-value		0.211		0.095		0.079			0.623
*Age*									
< 31	158	60.47 ± 13.25	159	14.16 ± 3.10	160	23.04 ± 4.89	159		23.26 ± 6.68
31–40	191	58.57 ± 13.99	198	14.04 ± 3.37	198	22.13 ± 5.24	193		22.30 ± 6.78
41–50	227	60.77 ± 13.83	246	14.20 ± 3.47	240	22.92 ± 5.41	236		23.60 ± 6.67
51–60	196	62.82 ± 13.07	218	14.50 ± 3.00	213	23.98 ± 4.97	204		24.29 ± 6.37
> 60	145	65.52 ± 12.79	156	15.31 ± 3.02	153	24.74 ± 4.75	149		25.11 ± 6.66
*p*-value		** < 0.001**		** < 0.001**		** < 0.001**			**0.002**
*Career stage*									
Chiropractic student	29	60.45 ± 13.86	29	13.72 ± 3.26	29	22.41 ± 4.92	29		24.31 ± 6.66
Currently practicing chiropractic	873	61.54 ± 13.59	931	14.42 ± 3.25	918	23.34 ± 5.16	897		23.69 ± 6.66
Other (including on leave, paused, not practicing, or retired)	19	56.95 ± 13.64	21	14.38 ± 3.12	21	22.10 ± 4.91	19		20.84 ± 6.82
*p*-value		0.303		0.433		0.229			0.170
*Area of practice*									
General chiropractic practice	760	61.48 ± 13.51	813	14.42 ± 3.24	800	23.27 ± 5.15	779		23.70 ± 6.64
Focused practice	101	61.46 ± 15.02	104	14.36 ± 3.52	105	23.74 ± 5.58	104		23.36 ± 7.17
Other (including administrative, research, etc.)	15	64.60 ± 8.46	15	15.07 ± 1.83	15	23.53 ± 3.16	15		26.00 ± 5.32
*p*-value		0.774		0.947		0.406			0.362
*Years in practice*									
5 or less years	187	59.55 ± 13.07	190	14.01 ± 3.18	191	22.81 ± 4.97	190		22.71 ± 6.49
6–10 years	105	58.26 ± 14.34	109	13.82 ± 3.63	109	21.87 ± 5.18	105		22.40 ± 6.89
11–15 years	98	59.69 ± 12.98	104	14.38 ± 3.21	104	22.85 ± 4.80	100		22.48 ± 6.49
16–20 years	88	59.91 ± 15.64	99	14.30 ± 3.67	94	22.57 ± 5.85	92		23.13 ± 7.57
21–25 years	142	61.85 ± 13.78	152	14.27 ± 3.24	150	23.13 ± 5.66	148		24.07 ± 6.33
26–30 years	102	64.26 ± 12.23	113	14.62 ± 2.99	111	24.50 ± 4.55	106		24.93 ± 6.19
31 years or more	157	65.73 ± 12.38	170	15.41 ± 2.74	166	25.04 ± 4.55	161		25.35 ± 6.54
*p*-value		** < 0.001**		** < 0.001**		** < 0.001**			** < 0.001**
*Community size*									
Urban or suburban	620	61.18 ± 13.65	663	14.33 ± 3.24	655	23.33 ± 5.19	633		23.42 ± 6.79
Small town or rural	249	62.50 ± 13.06	264	14.71 ± 3.20	260	23.43 ± 4.99	259		24.28 ± 6.23
Other (incl. mobile and virtual)	8	62.00 ± 15.90	9	14.11 ± 3.92	9	23.78 ± 5.19	8		24.88 ± 7.72
*p*-value		0.371		0.168		0.939			0.230
*Practice format*									
Community—interdisciplinary/medical	345	61.67 ± 13.03	370	14.44 ± 3.02	365	23.38 ± 4.94	353		23.75 ± 6.42
Community – solo/multi chiropractor practice	316	61.98 ± 13.73	337	14.51 ± 3.46	332	23.57 ± 5.29	325		23.84 ± 6.84
Community – interdisciplinary rehab	214	60.63 ± 14.05	227	14.30 ± 3.27	225	22.94 ± 5.22	220		23.31 ± 6.79
*p*-value		0.555		0.359		0.194			0.703
*Disability*									
No disability	725	62.37 ± 13.29	770	14.67 ± 3.14	757	23.57 ± 5.02	746		24.07 ± 6.57
With disability or mental health / chronic condition	182	57.67 ± 14.31	195	13.28 ± 3.49	194	22.09 ± 5.58	185		22.12 ± 6.88
*p*-value		** < 0.001**		** < 0.001**		** < 0.001**			** < 0.001**
*Caregiver status*									
Caregiver	378	62.26 ± 13.55	407	14.52 ± 3.26	397	23.36 ± 5.20	389		24.20 ± 6.66
Not a caregiver	542	60.83 ± 13.62	572	14.31 ± 3.24	569	23.24 ± 5.13	555		23.28 ± 6.66
*p*-value		0.099		0.207		0.526			**0.036**
*Payment sources*									
Direct out-of-pocket	394	63.41 ± 12.98	426	14.83 ± 3.18	419	24.21 ± 4.88	406		24.31 ± 6.62
Third-party payer	478	60.16 ± 13.81	503	14.10 ± 3.27	500	22.69 ± 5.25	487		23.22 ± 6.66
*p*-value		** < 0.001**		** < 0.001**		** < 0.001**			**0.013**

SD, standard deviation; MHC-SF, mental health continuum-short form

^*^This table reports mean scores and standard deviations for the MHC-SF full scale and subscales (emotional, psychological, and social well-being). Data are stratified by collapsed demographic and professional characteristics. *P*-values indicate statistical significance of group differences.

**Table 3:** Generalized Anxiety Disorder (GAD) and Maslach Burnout Inventory (MBI) Burnout Scores*

Characteristics	GAD (range 0–21)		MBI Full Scale (range 0–12)		MBI Emotional Exhaustion (range 0–6)		MBI Depersonalization (range 0–6)	
	n	Mean ± SD	n	Mean ± SD	n	Mean ± SD	n	Mean ± SD
All usable responses	1268	5.90 ± 5.34	1330	4.01 ± 3.13	1334	2.64 ± 1.82	1334	1.38 ± 1.66
*Gender*								
Man	639	5.04 ± 5.28	677	3.76 ± 3.12	679	2.42 ± 1.85	679	1.34 ± 1.64
Woman	612	6.79 ± 5.21	635	4.33 ± 3.13	636	2.88 ± 1.76	637	1.44 ± 1.70
Another gender	4	3.75 ± 4.92	4	2.25 ± 2.87	5	1.80 ± 2.68	4	0.00 ± 0.00
*p*-value		** < 0.001**		** < 0.001**		** < 0.001**		0.074
*Age*								
< 31	217	8.25 ± 5.41	224	5.65 ± 3.05	224	3.50 ± 1.59	224	2.15 ± 1.83
31–40	250	7.26 ± 5.48	263	4.67 ± 3.15	263	3.00 ± 1.77	264	1.67 ± 1.75
41–50	314	5.88 ± 5.12	320	3.93 ± 3.02	321	2.62 ± 1.77	322	1.31 ± 1.63
51–60	283	4.83 ± 4.79	293	3.58 ± 2.97	295	2.43 ± 1.80	294	1.16 ± 1.56
> 60	200	3.21 ± 4.55	224	2.28 ± 2.49	225	1.63 ± 1.71	224	0.64 ± 1.09
*p*-value		** < 0.001**		** < 0.001**		** < 0.001**		** < 0.001**
*Career stage*								
Chiropractic student	41	9.90 ± 5.89	42	6.83 ± 3.03	42	4.29 ± 1.67	42	2.55 ± 1.86
Currently practicing chiropractic	1199	5.76 ± 5.25	1255	3.92 ± 3.10	1259	2.58 ± 1.81	1259	1.34 ± 1.65
Other (including on leave, paused, not practicing, or retired)	28	6.11 ± 5.94	32	4.06 ± 2.83	32	2.63 ± 1.74	32	1.44 ± 1.63
*p*-value		** < 0.001**		** < 0.001**		** < 0.001**		** < 0.001**
*Area of practice*								
General chiropractic practice	1050	5.74 ± 5.30	1093	3.88 ± 3.10	1095	2.55 ± 1.81	1095	1.33 ± 1.64
Focused practice	138	6.01 ± 5.17	147	4.37 ± 3.16	148	2.86 ± 1.79	149	1.50 ± 1.76
Other (including administrative, research, etc.)	18	4.61 ± 4.17	22	3.05 ± 2.42	22	2.09 ± 1.51	22	0.95 ± 1.33
*p*-value		0.541		0.087		0.073		0.442
*Years in practice*								
5 or less years	255	7.83 ± 5.24	263	5.10 ± 3.02	263	3.16 ± 1.62	263	1.94 ± 1.76
6–10 years	132	7.29 ± 5.42	140	4.65 ± 3.05	140	3.09 ± 1.72	140	1.56 ± 1.71
11–15 years	134	6.22 ± 5.45	137	4.09 ± 3.06	137	2.64 ± 1.79	139	1.42 ± 1.65
16–20 years	128	5.47 ± 4.97	133	3.91 ± 3.11	133	2.59 ± 1.78	133	1.32 ± 1.72
21–25 years	199	5.39 ± 4.91	204	3.85 ± 2.94	205	2.63 ± 1.75	205	1.22 ± 1.56
26–30 years	141	5.02 ± 5.07	146	3.49 ± 3.20	148	2.34 ± 1.87	147	1.16 ± 1.66
31 years or more	221	3.22 ± 4.35	244	2.47 ± 2.58	245	1.72 ± 1.73	244	0.74 ± 1.19
*p*-value		** < 0.001**		** < 0.001**		** < 0.001**		** < 0.001**
*Community size*								
Urban or suburban	869	5.79 ± 5.23	911	3.86 ± 3.03	914	2.56 ± 1.77	915	1.31 ± 1.62
Small town or rural	327	5.80 ± 5.37	341	4.06 ± 3.20	342	2.64 ± 1.88	341	1.41 ± 1.70
Other (incl. mobile and virtual)	11	4.82 ± 5.38	12	3.50 ± 3.58	12	2.17 ± 2.12	12	1.33 ± 1.78
*p*-value		0.690		0.593		0.572		0.780
*Practice format*								
Community—interdisciplinary/medical	471	5.94 ± 5.11	484	3.95 ± 2.93	487	2.67 ± 1.74	484	1.28 ± 1.55
Community – solo/multi chiropractor practice	423	4.96 ± 5.16	453	3.50 ± 3.10	454	2.29 ± 1.84	455	1.21 ± 1.60
Community – interdisciplinary rehab	310	6.54 ± 5.46	324	4.41 ± 3.21	324	2.81 ± 1.79	326	1.59 ± 1.78
*p*-value		** < 0.001**		** < 0.001**		** < 0.001**		**0.010**
*Disability*								
No disability	991	5.45 ± 5.12	1045	3.84 ± 3.03	1047	2.53 ± 1.77	1049	1.32 ± 1.61
With disability or mental health / chronic condition	256	7.66 ± 5.79	263	4.68 ± 3.40	265	3.06 ± 1.97	263	1.61 ± 1.83
*p*-value		** < 0.001**		** < 0.001**		** < 0.001**		**0.033**
*Caregiver status*								
Caregiver	536	5.84 ± 5.04	562	3.88 ± 2.89	563	2.65 ± 1.74	564	1.24 ± 1.55
Not a caregiver	729	5.95 ± 5.56	764	4.12 ± 3.30	767	2.63 ± 1.89	766	1.48 ± 1.74
*p*-value		0.594		0.595		0.677		**0.028**
*Payment sources*								
Direct out-of-pocket	559	4.81 ± 4.69	591	3.40 ± 2.82	594	2.33 ± 1.76	593	1.07 ± 1.44
Third-party payer	642	6.61 ± 5.61	663	4.36 ± 3.24	664	2.80 ± 1.81	665	1.56 ± 1.77
*p*-value		** < 0.001**		** < 0.001**		** < 0.001**		** < 0.001**

SD, standard deviation; GAD, Generalized Anxiety Disorder; MBI, Maslach Burnout Inventory

^*^This table reports mean scores and standard deviations for GAD, and MBI (full scale, emotional exhaustion, and depersonalization). Data are stratified by collapsed demographic and professional characteristics. *P*-values indicate statistical significance of group differences.

**Table 4:** Mental health continuum-short form scale and subscale categories*

Characteristics	MHC-SF full scale				MHC-SF emotional subscale			MHC-SF social subscale			MHC-SF psychological subscale		
	n	Flourishing %	Moderate %	Languishing %	n	High %	Low %	n	High %	Low %	n	High %	Low %
All usable responses	922	57.8	36.9	5.3	982	95.5	4.5	946	93.7	6.3	969	90.2	9.8
*Gender*													
Man	454	63.4	30.6	5.9	489	95.7	4.3	467	93.8	6.2	478	89.7	10.3
Woman	459	52.5	42.7	4.8	481	95. 4	4.6	470	93.4	6.6	479	90.8	9.2
Another gender	2	50.0	50.0	0.0	3	100.0	0.0	2	100.0	0.0	3	100.0	0.0
*p*-value		**0.002**				0.891			0.906			0.698	
*Age*													
< 31	158	49.4	44.9	5.7	159	96.2	3.8	159	92.5	7.5	160	90.6	9.4
31–40	191	46.6	47.1	6.3	198	94.4	5.6	193	90.7	9.3	198	86.4	13.6
41–50	227	58.6	34.4	7.0	246	94.3	5.7	236	92.8	7.2	240	89.6	10.4
51–60	196	66.3	28.6	5.1	218	96.3	3.7	204	96.1	3.9	213	91.5	8.5
> 60	145	70.3	28.3	1.4	156	97.4	2.6	149	96.6	3.4	153	94.1	5.9
*p*-value		** < 0.001**				0.513			0.100			0.161	
*Career stage*													
Chiropractic student	29	44.8	44.8	10.3	29	93.1	6.9	29	93.1	6.9	29	86.2	13.8
Currently practicing chiropractic	873	58.6	36.3	5.0	931	95.6	4.4	897	93.9	6.1	918	90.4	9.6
Other (including on leave, paused, not practicing, or retired)	19	42.1	47.4	10.5	21	95.2	4.8	19	84.2	15.8	21	90.5	9.5
*p*-value		0.141				0.444			0.157			0.685	
*Area of practice*													
General chiropractic practice	760	58.6	36.7	4.7	813	95.6	4.4	779	93.7	6.3	800	90.6	9.4
Focused practice	101	57.4	34.7	7.9	104	94.2	5.8	104	93.3	6.7	105	87.6	12.4
Other (including administrative, research, etc.)	15	66.7	33.3	0.0	15	100.0	0.0	15	100.0	0.0	15	93.3	6.7
*p*-value		0.651				0.736			0.810			0.621	
*Years in practice*													
5 or less years	187	49.2	47.1	3.7	190	95.3	4.7	90	93.2	6.8	191	91.1	8.9
6–10 years	105	49.5	41.9	8.6	109	92.7	7.3	105	90.5	9.5	109	87.2	12.8
11–15 years	98	51.0	44.9	4.1	104	94.2	5.8	100	91.0	9.0	104	91.3	8.7
16–20 years	88	55.7	33.0	11.4	99	94.9	5.1	92	89.1	10.9	94	83.0	17.0
21–25 years	142	62.7	30.3	7.0	152	96.1	3.9	148	94.6	5.4	150	88.7	11.3
26–30 years	102	63.7	34.3	2.0	113	96.5	3.5	106	100.0	0.0	111	94.6	5.4
31 years or more	157	73.9	24.2	1.9	170	98.2	1.8	161	95.7	4.3	166	94.0	6.0
*p*-value		** < 0.001**				0.367			**0.022**			**0.048**	
*Community size*													
Urban or suburban	620	57.1	37.9	5.0	663	95.5	4.5	633	93.0	7.0	655	90.8	9.2
Small town or rural	249	62.2	33.3	4.4	264	96.6	3.4	259	95.8	4.2	260	90.4	9.6
Other (incl. mobile and virtual)	8	50	37.5	12.5	9	88.9	11.1	8	100.0	0.0	9	88.9	11.1
*p*-value		0.400				0.307			0.291			0.777	
*Practice format*													
Community—interdisciplinary/medical	345	59.1	36.5	4.3	370	96.8	3.2	353	94.9	5.1	365	91.2	8.8
Community—solo/multi chiropractor practice	316	58.5	36.7	4.7	337	94.4	5.6	325	94.2	5.8	332	90.7	9.3
Community—interdisciplinary rehab	214	57	36.9	6.1	227	96.0	4.0	220	91.8	8.2	225	89.3	10.7
*p*-value		0.919				0.281			0.315			0.743	
*Disability*													
No disability	725	60.3	35.3	4.4	770	96.5	3.5	746	94.6	5.4	757	91.7	8.3
With disability or mental health / chronic condition	182	50.5	40.1	9.3	195	91.3	8.7	185	89.2	10.8	194	84.0	16.0
*p*-value		**0.007**				**0.003**			**0.011**			**0.002**	
*Caregiver status*													
Caregiver	378	61.1	33.9	5	407	95.1	4.9	389	94.6	5.4	397	89.7	10.3
Not a caregiver	542	55.7	38.7	5.5	572	95.8	4.2	555	93.0	7.0	569	90.7	9.3
*p*-value		0.263				0.705			0.382			0.680	
*Payment sources*													
Direct out-of-pocket	394	63.5	33.0	3.6	426	96.5	3.5	406	94.8	5.2	419	92.6	7.4
Third-party payer	478	54.8	39.1	6.1	503	95.0	5.0	487	93.4	6.6	500	89.0	11.0
*p*-value		**0.021**				0.357			0.460			0.080	

MHC-SF, mental health continuum-short form

^*^This table categorizes respondents’ mental health and well-being using the Mental Health Continuum-Short Form (MHC-SF). Results are stratified by collapsed demographic and professional characteristics. Categories include flourishing, moderate, and languishing mental health, as well as levels of anxiety, burnout, emotional exhaustion, and depersonalization. *P*-values indicate statistical significance of group differences.

**Table 5:** Generalized Anxiety Disorder (GAD) and Maslach Burnout Inventory (MBI) Burnout Categories*

Characteristics	GAD					MBI Full Scale			MBI Emotional Exhaustion			MBI Depersonalization		
	n	Minimal %	Mild %	Moderate %	Severe %	n	High %	Low %	n	High %	Low %	n	High %	Low %
All usable responses	1268	47.9	30.1	13.1	8.9	1332	33.2	66.8	1334	31.5	68.5	1334	13.2	86.8
*Gender*														
Man	639	56.7	26.1	9.5	7.7	679	30.2	69.8	679	28.3	71.7	679	12.2	87.8
Woman	612	38.7	34.6	16.5	10.1	635	36.9	63.1	636	35.4	64.6	637	14.6	85.4
Another gender	4	75.0	0.0	25.0	0.0	4	25.0	75.0	5.0	20.0	80.0	4	0.0	100.0
*p*-value		** < 0.001**					**0.030**			**0.014**			0.366	
*Age*														
< 31	217	27.6	36.4	21.2	14.7	224	50.0	50.0	224	48.7	51.3	224	25.0	75.0
31–40	250	34.8	36.8	17.2	11.2	263	39.5	60.5	263	36.9	63.1	264	17.0	83.0
41–50	314	45.9	33.1	12.4	8.6	321	30.8	69.2	321	29.3	70.7	322	10.9	89.1
51–60	283	58.3	26.9	7.8	7.1	294	28.9	71.1	295	27.1	72.9	294	11.2	88.8
> 60	200	75.0	14.5	7.5	3.0	224	17.9	82.1	225	16.9	83.1	224	2.7	97.3
*p*-value		** < 0.001**					** < 0.001**			** < 0.001**			** < 0.001**	
*Career stage*														
Chiropractic student	41	24.4	24.4	26.8	24.4	42	71.4	28.6	42	69.0	31.0	42.0	38.1	61.9
Currently practicing chiropractic	1199	48.7	30.2	12.8	8.3	1257	32.1	67.9	1259	30.4	69.6	1259	12.5	87.5
Other (including on leave, paused, not practicing, or retired)	28	46.4	35.7	3.6	14.3	32	25.0	75.0	32	25.0	75.0	32	9.4	90.6
*p*-value		** < 0.001**					** < 0.001**			** < 0.001**			** < 0.001**	
*Area of practice*														
General chiropractic practice	1050	49.1	29.9	12.4	8.6	1094	31.5	68.5	1095	30.0	70.0	1095	12.1	87.9
Focused practice	138	44.9	33.3	13.8	8.0	148	37.2	62.8	148	34.5	65.5	149	16.1	83.9
Other (including administrative, research, etc.)	18	55.6	27.8	16.7	0.0	22	22.7	77.3	22	22.7	77.3	22	9.1	90.9
*p*-value		0.823					0.248			0.393			0.363	
*Years in practice*														
5 or less years	255	29.0	38.8	20.8	11.4	263	43	57	263	41.1	58.9	263	20.2	79.8
6–10 years	132	31.1	42.4	14.4	12.1	140	39.3	60.7	140	36.4	63.6	140	15.7	84.3
11–15 years	134	46.3	32.1	11.2	10.4	137	33.6	66.4	137	31.4	68.6	139	12.9	87.1
16–20 years	128	50.8	28.9	11.7	8.6	133	30.8	69.2	133	29.3	70.7	133	12.0	88.0
21–25 years	199	50.3	29.6	14.1	6.0	205	30.2	69.8	205	29.3	70.7	205	9.8	90.2
26–30 years	141	57.4	26.2	7.8	8.5	147	28.6	71.4	148	27.0	73.0	147	13.6	86.4
31 years or more	221	74.2	16.3	6.3	3.2	244	19.3	80.7	245	18.0	82.0	244	3.7	96.3
*p*-value		** < 0.001**					** < 0.001**			** < 0.001**			** < 0.001**	
*Community size*														
Urban or suburban	869	48.1	31.1	12.7	8.2	913	30.4	69.6	914	29.0	71.0	915	11.6	88.4
Small town or rural	327	49.2	29.4	11.9	9.5	341	36.1	63.9	342	33.6	66.4	341	14.4	85.6
Other (incl. mobile and virtual)	11	54.5	18.2	27.3	0.0	12	33.3	66.7	12	33.3	66.7	12	8.3	91.7
*p*-value		0.706					0.157			0.268			0.394	
*Practice format*														
Community—interdisciplinary/medical	471	46.9	31.6	12.7	8.7	485	33.0	67.0	487	31.8	68.2	484	10.1	89.9
Community—solo/multi chiropractor practice	423	55.1	27.9	10.2	6.9	454	26.7	73.3	454	24.2	75.8	455	11.4	88.6
Community—interdisciplinary rehab	310	42.9	31.9	15.2	10.0	324	37.3	62.7	324	35.8	64.2	326	16.6	83.4
*p*-value		**0.041**					**0.005**			**0.001**			**0.019**	
*Disability*														
No disability	991	51.5	29.4	11.7	7.5	1047	30.3	69.7	1047	28.5	71.5	1049	11.8	88.2
With disability or mental health / chronic condition	256	34.4	32.8	18.4	14.5	263	44.9	55.1	265	43.4	56.6	263	18.6	81.4
*p*-value		** < 0.001**					** < 0.001**			** < 0.001**			**0.005**	
*Caregiver status*														
Caregiver	536	46.3	33.2	12.5	8.0	563	31.3	68.7	563	29.1	70.9	564	10.8	89.2
Not a caregiver	729	49.2	27.6	13.6	9.6	765	34.8	65.2	767	33.4	66.6	766	15.0	85.0
p-value		0.173					0.200			0.113			**0.031**	
*Payment sources*														
Direct out-of-pocket	559	54.6	29.7	10.7	5.0	592	27.0	73.0	594	25.6	74.4	593	7.8	92.2
Third-party payer	642	43.3	31.00	14.2	11.5	668	36.4	63.6	668	34.4	65.6	669	16.4	83.6
*p*-value		** < 0.001**					** < 0.001**			** < 0.001**			** < 0.001**	

GAD, generalized anxiety disorder; MBI, Maslach burnout inventory

^*^This table categorizes respondents’ Generalized Anxiety Disorder (GAD) scale, and Maslach Burnout Inventory (MBI) full scale and subscales. Results are stratified by collapsed demographic and professional characteristics. Categories include flourishing, moderate, and languishing mental health, as well as levels of anxiety, burnout, emotional exhaustion, and depersonalization. P-values indicate statistical significance of group differences.

**Table 6:** PHQ-2 and suicidal ideation*

	PHQ-2 Anhedonia (Lifetime)		PHQ-2 Depressed mood (Lifetime)		Suicidal ideation (Recent)		Suicidal ideation (Lifetime)	
	n	Yes %	n	Yes %	n	Yes %	n	Yes %
All usable responses	1270	41.3	1271	36.8	323	38.7	1152	26.7
*Gender*								
Man	646	37.3	646	31.0	163	41.1	570	26.7
Woman	607	45.6	608	43.1	154	35.1	571	26.4
Another gender	4	25.0	4	25.0	1	100.0	3	33.3
*p*-value		**0.005**		** < 0.001**		0.197		0.976
*Age*								
< 31	213	53.5	213	49.8	67	34.3	198	33.8
31–40	249	52.2	249	42.2	69	40.6	238	30.3
41–50	307	42.3	307	39.7	65	52.3	283	23.3
51–60	284	33.5	285	29.8	82	36.6	253	29.6
> 60	212	24.5	212	22.6	38	23.7	177	14.7
*p*-value		** < 0.001**		** < 0.001**		**0.050**		** < 0.001**
*Career stage*								
Chiropractic student	40	67.5	40	55.0	14	50.0	37	35.1
Currently practicing chiropractic	1199	40.0	1200	36.2	299	38.5	1092	26.2
Other (including on leave, paused, not practicing, or retired)	30	53.3	30	36.7	10	30.0	23	39.1
*p*-value		** < 0.001**		0.052		0.586		0.192
*Area of practice*								
General chiropractic practice	1043	39.9	1044	35.5	253	36.0	950	25.6
Focused practice	139	44.6	139	39.6	41	46.3	125	31.2
Other (including administrative, research, etc.)	22	22.7	22	36.4	4	25.0	18	11.1
*p*-value		0.138		0.648		0.458		0.154
*Years in practice*								
5 or less years	250	51.6	250	48.8	73	31.5	234	32.5
6–10 years	133	48.1	133	39.8	33	57.6	125	26.4
11–15 years	133	48.9	133	39.8	35	34.3	129	28.7
16–20 years	126	40.5	126	41.3	24	54.2	110	20.0
21–25 years	192	39.6	193	33.7	50	40.0	178	29.2
26–30 years	144	29.2	144	26.4	40	37.5	125	27.2
31 years or more	233	24.5	233	23.2	46	23.9	201	16.9
*p*-value		** < 0.001**		** < 0.001**		**0.035**		**0.008**
*Community size*								
Urban or suburban	869	40.5	871	36.9	213	36.6	789	25.6
Small town or rural	329	38.3	328	34.1	85	38.8	303	27.1
Other (incl. mobile and virtual)	11	54.5	11	36.4	3	33.3	8	37.5
*p*-value		0.462		0.668		0.907		0.575
*Practice format*								
Community—interdisciplinary/medical	466	40.6	467	33.4	111	33.3	427	25.3
Community—solo/multi chiropractor practice	431	35.5	431	34.8	115	40.0	389	26.7
Community—interdisciplinary rehab	308	45.5	308	41.9	70	37.1	281	25.3
*p*-value		**0.023**		**0.044**		0.582		0.871
*Disability*								
No disability	998	37.4	999	31.9	214	36.9	913	22.5
With disability or mental health / chronic condition	253	55.7	253	55.7	106	42.5	222	45.0
*p*-value		** < 0.001**		** < 0.001**		0.404		** < 0.001**
*Caregiver status*								
Caregiver	530	40.9	530	35.7	117	41.9	488	22.7
Not a caregiver	736	41.3	737	37.7	206	36.9	661	29.8
*p*-value		0.944		0.490		0.444		**0.009**
*Payment sources*								
Direct out-of-pocket	569	34.8	570	32.6	140	33.6	512	25.4
Third-party payer	630	44.2	630	39.2	158	41.1	583	26.6
*p*-value		**0.001**		**0.021**		0.220		0.703

PHQ-2, 2-item version of the Patient Health Questionnaire (PHQ) depression module

^*^This table summarizes responses to screening questions on 2 items of PHQ-2 (lifetime anhedonia, lifetime depressed mood) and suicidal ideation (recent and lifetime). Results are presented by collapsed demographic and professional subgroups, with *p*-values indicating statistical differences across groups.

### MHC-SF

The Mental Health Continuum–Short Form (MHC-SF) was evaluated as both a continuous and categorical measure to assess overall mental well-being and its emotional, psychological, and social subdomains among chiropractic professionals. Among valid responses, the mean overall MHC-SF score was 61.4, with subscale means of 14.4 for emotional well-being, 23.7 for social well-being, and 23.3 for psychological well-being. When categorized, 57.8% of respondents were classified as having flourishing mental health, while 5.3% were categorized as languishing. High levels of the subdomain, well-being, were common: 95.5% met the criteria for high emotional well-being, 93.7% for high social well-being, and 90.2% for high psychological well-being.

Consistent patterns emerged across both scaling approaches: higher well-being was associated with an absence of a disability or chronic mental health condition, and direct out-of-pocket payment sources. These same groups showed higher proportions of individuals classified as flourishing. Respondents with a disability or chronic mental health condition had significantly lower mean scores across all domains and were less likely to be classified as flourishing.

However, some discrepancies were observed. For example, older respondents and longer years in practice were progressively associated with increased mean scores for the full scale and across all sub-domains, while categorical classification only showed a significant difference among age groups for the overall mental well-being but not for any of the sub-domains. Further, years in practice did not show significant differences for emotional well-being when using High/Low classification. While the continuous scores suggested no significant gender differences, the categorical scale showed men had significantly higher flourishing rates than women (*p* = 0.002).

Gender identity (except in full scale categorization), career stage, area of practice, community size, and practice format were non-significant factors, suggesting that in our sample, mental well-being is not strongly influenced by these variables in this sample.

### Anxiety

Among valid responses, the mean overall anxiety score was 5.9 (of the 21 maximum), and 22% of participants were classified as having moderate to severe levels of anxiety. Anxiety analyzed in both the continuous scale (mean anxiety scores) and the categorical classification (anxiety severity levels) revealed a high degree of concordance across several key respondent characteristics. Specifically, significant differences were observed by gender, age, years in practice, career stage, practice format, disability status, and payment source. Women reported significantly higher mean anxiety scores compared to men and were more likely to be categorized in the severe anxiety group. Similarly, anxiety levels decreased progressively with age and years of professional practice in both declining mean scores and the decreasing proportion of respondents classified as having moderate to severe anxiety. Respondents reporting a disability or chronic mental health condition showed elevated anxiety across both scales. Chiropractic students exhibited higher mean anxiety scores and a higher percentage in the severe and moderate anxiety groups than chiropractors currently in practice.

Solo or multi-chiropractor practices were associated with lower mean anxiety scores and a smaller proportion in the mild to severe anxiety category compared to other practice formats, while community interdisciplinary rehabilitation settings showed higher anxiety levels than other practice formats. Additionally, those with third-party payer models for care demonstrated higher anxiety levels than those relying primarily on out-of-pocket payments.

Several respondent characteristics, including area of practice, caregiver status, and community size (urban/suburban vs. rural or other), were not statistically significantly associated with anxiety levels.

### Burnout

Among valid responses, the mean overall MBI Burnout composite score was 4.0, with subscale means of 2.6 for MBI-Emotional Exhaustion (MBI-EE) and 1.4 for MBI-Depersonalization (MBI-DP). When the risk levels were categorized, 33.2% of participants were at high risk for overall burnout, 31.5% at high risk for emotional exhaustion, and 13.2% were at high risk for depersonalization. The Overall MBI Burnout composite score and MBI-EE subscale track closely and consistently identify key at-risk subgroups, such as younger (particularly marked in the < 31 age group), less experienced, female, student, identified as disabled, and third-party payer arrangement, as shown by both continuous and categorical burnout measures. The MBI-DP scale generally had lower mean values and smaller effect sizes, indicating it may be less sensitive in this population compared to MBI-EE. For example, women reported significantly higher burnout on the overall score and MBI-EE, but not on MBI-DP, suggesting that emotional exhaustion drives most group differences. For practice format, though it was significant with all three burnout measures, interdisciplinary rehabilitation settings had the highest burnout across all three measures by both continuous and categorical scales, with more pronounced differences in MBI-EE and smaller effects observed on MBI-DP. However, caregiver status showed no difference in overall burnout score or MBI-EE, but a significant difference in MBI-DP, suggesting depersonalization might be more tied to caregiving. No significant differences in burnout scores were detected across community size or area of practice categories in any measure.

### Depression

Lifetime anhedonia (PHQ-2.1) was reported by 41.3% of respondents overall and was significantly more common among women (45.6%) than men (37.3%, *p* = 0.005), and among those under the age of 40 years (*p* < 0.001). The highest rates were observed among chiropractic students (67.5%), while among practicing chiropractors, prevalence was highest in those with five or fewer years in practice (51.6%). Respondents with a disability or chronic condition were significantly more likely to report anhedonia (55.7%) than those without (37.4%, *p* < 0.001). Those working in interdisciplinary rehabilitation settings and those relying on third-party payer models also showed significantly higher prevalence compared to other practice formats and payment structures. Other factors, such as area of practice, community size, and caregiver status, did not significantly impact the likelihood of reporting anhedonia.

Lifetime depressed mood (PHQ-2.2) was reported by 36.8% of respondents overall and followed a pattern similar to anhedonia. It was significantly more prevalent among women (43.1%) than men (31.0%, *p* < 0.001), and most common among the youngest age group (< 31 years, 49.8%). Chiropractic students reported the highest prevalence (55.0%). While career stage strongly differentiated reports of anhedonia (*p* < 0.001), the significant association was not observed for depressed mood (*p* = 0.052). Among practicing chiropractors, the prevalence of depressed mood decreased steadily with increasing years in practice (*p* < 0.001). Respondents with a disability or chronic condition were significantly more likely to report depressed mood than those without (55.7% vs. 31.9%, *p* < 0.001). Higher prevalence was also observed in interdisciplinary rehabilitation settings (*p* = 0.044) and among those using third-party payer arrangements (*p* = 0.021). No significant differences were observed by area of practice, community size, or caregiver status.

### Recent suicidal ideation

Only 15% of respondents chose to answer the question regarding feelings of recent suicidal ideation, of whom 38.7% reported having such feelings. Age and years in practice were significantly associated with recent suicidal thoughts. Respondents aged 41–50 years had the highest prevalence (52.3%, *p* = 0.05), and elevated rates were also observed among those with 6–10 years (57.6%) and 16–20 years (54.2%) in practice (*p* = 0.035). No other demographic or practice-related characteristics, including gender, career stage, practice format, or disability status, were significantly associated with recent suicidal ideation.

### Lifetime suicidal ideation

Almost 54% of respondents answered the question regarding lifetime suicidal ideation, of which 26.7% responded in the affirmative. Age was significantly associated with lifetime suicidal ideation (*p* < 0.001), with the highest prevalence observed among the youngest respondents (< 31 years) and those aged 51–60, while the oldest age group (> 60 years) had the lowest rate. Years in practice also demonstrated a significant association (*p* = 0.008), with elevated rates among those practicing ≤ 5 years and those with 21–25 years in practice, and the lowest rates among those with ≥ 31 years in practice. Respondents reporting a disability or chronic condition had substantially higher rates of lifetime suicidal ideation compared to those without (45.0% vs. 22.5%, *p* < 0.001). Interestingly, caregivers reported significantly lower rates than non-caregivers (22.7% vs. 29.8%, *p* = 0.009). No significant differences were observed by gender, community size, area of practice, or payment source.

## Discussion

This study is the first comprehensive evaluation of mental health and wellness in the Canadian chiropractic profession. In general, participating chiropractors reported being in relatively good well-being and mental health, with over 90% reporting high emotional, social and psychological well-being, and 57.8% having flourishing and 5.3% languishing mental health. Similarly, the majority reported minimal to mild anxiety (78%) and low burnout (66.8%). Lifetime anhedonia and depressed mood were reported by 41.3% and 36.8% of participants, respectively. Of the 54% of participants who responded, 26.7% answered affirmatively to lifetime suicidal ideation, but only 15% of participants chose to respond to the question related to recent ideation, of which 38.7% answered yes. There are important caveats across each psychological measure in terms of the distribution and differences in scores between sub-groups, some resonating with data from health profession research.

Research focusing on the psychological well-being of the chiropractic profession has primarily addressed burnout. Partridge et al. [13] summarized the results of 3 cross-sectional studies conducted in the US of discrete groups of chiropractors. They report that studies found lower levels of burnout as measured using the MBI compared to medical, nursing and other health professions. Williams and Zip [21] reported on one of the largest studies (n = 1162) assessing burnout among a convenience sample of practicing chiropractors. They reported that 20% of participants had high emotional exhaustion and 8% had high depersonalization, and women had significantly lower DP scores than men. These findings differ from our study, wherein we reported higher values in both categories and a significant gender difference was noted only for overall burnout and emotional exhaustion. Our findings are similar to those for physicians, where overall burnout is significantly higher among women (59%) than men (43%) [9].

Similar to other healthcare professions [9, 10], the chiropractic profession also experiences mental health challenges. However, there were some notable differences when comparing the results from the Canadian chiropractic profession, medical profession and the public. A greater percentage of chiropractors (58%) were categorized as flourishing compared to their physician counterparts (47%) and those from the public (38%) [9]. Similarly, chiropractors had the smallest percentage (5%) of languishing members when compared to physicians (7%) and public (12%). Chiropractors also displayed less overall burnout compared to physicians, a little less anxiety, but the same percentage of members who screened positive for depression [9]. Similar to their physician counterparts, female chiropractors reported higher levels of anxiety and depression [9]. Our findings provide an opportunity to further explore how the chiropractor’s role in healthcare, gender and personal factors and practice characteristics may influence mental health.

Neither practice specialization nor the geographic location was significantly correlated with well-being; however, the choice of practice composition did have important implications. Chiropractors in a single profession, community practice (solo or group) had significantly less anxiety, burnout, and anhedonia compared to those in interprofessional settings. These results align with the rich body of research on the challenges associated with offering interprofessional care in Canada [34–37].

In our study, approximately 20% of respondents identified having a disability, most commonly mental health and chronic conditions. The pronounced effect of this reported disability status across nearly all mental health outcomes highlights the importance of inclusive and supportive work environments that accommodate physical and psychological health needs. Self-employed persons with disabilities working in Canada have been shown to have lower earnings with variations depending on the nature of their disability [38]. Additionally, they have limited access to social support systems available in Canada [39].

Third-party payment models were consistently associated with higher levels of anxiety, burnout, anhedonia, and depressed mood among chiropractic professionals. This may reflect the administrative burden and emotional toll of managing reimbursement processes and navigating payer requirements [40]. Future research could explore whether training dedicated billing personnel or implementing technologies that help with payment administration could reduce the direct burden on providers and impact overall mental health and well-being.

Previous studies of students attending a Canadian chiropractic program reported a two-week prevalence of depression of 23.6%, with differences among gender and years of study [41]. More recently, Meckamalil et al. [42] reported one-week prevalence of moderate to extreme symptoms of 19.0% (95% CI: 13.0–25.0) for depression; 32.6% (95% CI: 24.7–40.3) for anxiety and 21.8% (95% CI: 15.6–28.1) for stress, with higher scores for females and those in second year. We found higher rates of anhedonia and depressed mood among student participants, 67.5% and 55%, respectively, and 51.2% reported moderate to severe anxiety. Our findings were notably higher; however, our student response rate was low (8% response rate), and hence caution should be exercised when reporting and interpreting our results. Future work should aim to improve response rates.

Of greatest concern was the participants’ reporting recent and lifetime suicidal ideation. Acknowledging the lower response rates to the survey questions about recent (15%) and lifetime (54%) suicidal ideation, it was disconcerting that 38.7% and 26.7% of those responding identified positively to recent and lifetime suicidal ideation, respectively. These values are considerably higher than the 14% for recent but lower than the 36% lifetime suicidal ideation reported by physician colleagues [8]. Further research is needed into the potential reasons for the contemplation of suicide among chiropractors in Canada. These insights will be valuable as any suggestion of suicidal ideation requires available resources and interventions in the short term, and preventative strategies in the long term. Designing these interventions, and ultimately assessing their impact, will depend on how well we understand the causes and modifiers of suicidal ideation.

### Implications for professional associations

National, provincial or state professional associations and regulatory bodies serve multiple roles. The establishment of a professional association is the second stage of occupational development, occurring right after the creation of full-time work [43]. The association facilitates social recognition of an occupation as a profession in the public and legal realm [44]. Professions are built around a substantial body of knowledge; professional associations provide a milieu for expanding and revising their knowledge [45]. They also serve as social networks of people with similar interests, solidifying their members’ professional identity and belonging and offer access to relevant products and services [46, 47]. Members also have an expectation that their needs will be addressed, likely leading to further engagement with their association [47].

Our study identifies several areas of need among chiropractors in Canada linked to roles of associations. As the national chiropractic advocacy association in Canada, the CCA can lead the development and dissemination of evidence-informed interventions that specifically target its members. Our study findings suggest that mental health support initiatives should prioritize practitioners who are women, in the early stages of their career, students, and those working in interdisciplinary or third-party-dominated care environments, where emotional demands and systemic constraints may be highest. A recent systematic review identified several effective interventions to manage the symptoms of healthcare workers’ mental health challenges, including mindfulness, coping skills development, and relaxation and reflection [48]. An integration of these approaches has a positive impact on burnout, stress, depression, emotional exhaustion, and general well-being, as demonstrated in a recent systematic review [48]. Through the CCA’s learning management system, asynchronous, on-demand courses on mindfulness strategies and coping skills are currently available to members and will continue to be developed.

Another strategy shown to be effective in reducing burnout in healthcare providers is mentorship. Fagan et al. [49] define mentorship as a relationship whereby a more experienced or knowledgeable person (mentor) guides a less experienced or knowledgeable person (mentee) with the focus on developing the mentee’s ability to navigate challenges rather than acquiring specific knowledge or technical skills. Such an approach has been shown to combat burnout and benefit both mentors and mentees. In 2024, the CCA launched a pilot mentorship program to gather insights and feedback from its members. As a result of such findings, a fulsome mentorship program has launched in 2025 with pairings of mentees (0–9 years in practice) with a mentor (over 10 years in practice). In consideration of our study’s findings, future work will also focus on integrating chiropractic students into this program, with assistance from chiropractic institutions and the Student Canadian Chiropractic Association (SCCA), to support their mental health and well-being and cultivate community during their academic program. Further research aimed at identifying the causes of burnout, anxiety, or poor psychological well-being is needed to better address these challenges at their roots.

### Limitations

First, a cross-sectional design precludes any causal inferences between respondent characteristics and mental health outcomes. While associations are reported, the temporal relationships remain unclear. Second, the data were derived from a convenience sample of chiropractic professionals, which may limit generalizability. Individuals experiencing higher levels of distress may have been more motivated to respond, introducing self-selection bias. The response rate of 26% for chiropractors was higher than the usual 10% but lower than the 41% previously conducted CCA surveys [23]. Also, the student response rate of 8% was considerably lower than the 67% previously reported chiropractic student survey [42]. Strategies to improve response rates, including providing further information, assessing the timing of survey distribution and greater engagement of stakeholders should be considered. In addition, the sample was predominantly composed of white respondents, which is consistent with demographic information reported in the CCA Resources Databank 2022 Survey (84% of respondents identified as white); however, may not adequately reflect the experiences of individuals from racial and ethnic minority groups, limiting the applicability of findings to more diverse populations. Third, all data were self-reported, including sensitive measures such as depression, suicidal ideation and mental health symptoms, which may be subject to recall bias or social desirability bias. While validated instruments (e.g., PHQ-2, MBI, MHC-SF) were used to enhance reliability, no clinical diagnostic assessments were conducted, meaning that the reported mental health symptoms were not clinically confirmed. Fourth, missing data on some items (notably suicidal ideation) reduced the sample size for specific analyses and may affect the precision of prevalence estimates. Moreover, the missingness was not evenly distributed across all respondent subgroups, which could introduce bias in both prevalence estimates and subgroup comparisons, potentially overstating or understating associations in certain populations. Finally, this study is descriptive in nature and examined only bivariate associations between respondent characteristics and mental health outcomes. While this approach offers initial insights into potential correlates, it does not account for interactions or confounding effects between variables. The study did not collect potentially confounding variables, such as personal history of mental illness, social support, or workload, which could influence both mental health outcomes and professional characteristics.

## Conclusion

This is the first comprehensive evaluation of mental health and wellness in the chiropractic profession globally. The results demonstrate that, like other professions, there are mental health and wellness challenges in the Canadian chiropractic profession. Our results offer guidance for the targeting of intervention strategies for those most at-risk in an attempt to support the most vulnerable with limited resources. Further research is ongoing to better understand how well-being and mental health among chiropractors in Canada compare to their counterparts in other countries, and how these outcomes are affected by occupational circumstances, behavioural factors, and social supports.

## Supplementary Information

Below is the link to the electronic supplementary material.

Supplementary Material 1

Supplementary Material 2

## Data Availability

The data that support the findings of this study are available from the authors upon reasonable request. Some ethical restrictions may apply to the sharing of data to ensure that participants cannot be identified.
