# Supplementary material for: Psychological well-being among Canadian chiropractors: a cross-sectional questionnaire-based study
Source: Chiropr Man Therap. 2025 Nov 28;33:55. doi: 10.1186/s12998-025-00616-w (PMC12664135; doi:10.1186/s12998-025-00616-w)
Supplement: Supplementary file 1 — Supplementary Material 1 [file 12998_2025_616_MOESM1_ESM.docx]

**Additional file 1:** Questionnaire items assessing outcome variables

| **Questions** | **Response options** |
| --- | --- |
| *Mental health and well-being short form (MHC-SF)^24^* | |
| How often in the past month did you feel…   - Happy - Interested in life - Satisfied with your life - That you had something important to contribute to society - That you belonged to a community (e.g. social group, your neighbourhood, your city, your school) - That our society is becoming a better place for people like you - That people are basically good - That the way our society works makes sense to you - That you liked most parts of your personality - Good at managing the responsibilities of your daily life - That you had warm and trusting relationships with others - That you had experiences that challenged you to grow and become a better person - Confident to thing or express your own ideas and opinions - That your life had a sense of direction or meaning to it | 5 - Everyday  4 - Almost everyday  3 - About two or three times a week  2 - About once a week  1 - Once or twice  0 - Never |
| *Generalized Anxiety Disorder (GAD-7)^28^* | |
| How often have you been bothered by the following over the past two (2) weeks?   - Feeling nervous, anxious, or on edge - Not being able to stop or control worrying - Worrying too much about different things - Trouble relaxing - Being so restless that it’s hard to sit still - Becoming easily annoyed or irritable - Feeling afraid as if something awful might happen | 3 - Nearly every day  2 - More than half the days  1 - Several days  0 - Not at all |
| *Maslah Burnout Inventory (MBI) – 2 items^29^* | |
| - I feel burned out from my work or training environment - I have become more callous towards people since I took this job or started this training | 6 - Everyday  5 - A few times a week  4 - Once a week  3 - A few times a month  2 - Once a month or less  1 - A few times a year  0 – Never |
| *Depression (PHQ-2)^31^* | |
| - Was there ever a time lasting two weeks or more when you lost interest or pleasure in most things like hobbies and/or work activities that usually give you pleasure? - Was there ever a time when you felt down, depressed, or hopeless for two or more weeks in a row? | 1 – yes  0 – no |
| *Suicidal ideation* | |
| - Have you had thoughts of suicide? | 1 – yes, before my chiropractic training or education  1 – yes, during my training/ education  1 – yes, during chiropractic practice  0 – no  Prefer not to answer |
| - Have you had thoughts of suicide in the last 12 months? | 1 – yes  0 – no  Prefer not to answer |
