# Supplementary material for: Psychological well-being among Canadian chiropractors: a cross-sectional questionnaire-based study
Source: Chiropr Man Therap. 2025 Nov 28;33:55. doi: 10.1186/s12998-025-00616-w (PMC12664135; doi:10.1186/s12998-025-00616-w)
Supplement: Supplementary file 2 — Supplementary Material 2 [file 12998_2025_616_MOESM2_ESM.docx]

**Additional file 2:** Adjustments made to categories of respondent characteristics for purposes of sub-group comparisons

1. "Prefer not to say" and "Unknown" responses were not included in the group comparisons.
2. Ethnicity was not included in group comparisons due to the predominance of white respondents. Some respondent characteristics were consolidated to ensure sufficient sample size for group comparisons while maintaining the similarity of meanings.
3. "Trans man" was grouped with "Man" and "Trans woman" was grouped with "Woman", any other non-unknown gender responses were grouped as Another gender.
4. Disability and caregiver status were categorized as "Yes" or "No" response with multiple selects were grouped as "Yes". For career stage, area of practice, practice format/setting, and primary payment source, "Other (please specify) " responses were reviewed and redistributed to appropriate existing response categories where possible.
5. For career stage, "Currently practicing chiropractic" and "Chiropractic student" were kept as their own original categories; a new collapsed "Other" category was created to include "Graduate school student", "Clinical resident", "On leave from practice", "Paused practice but not retried", "Retired from practice".
6. For Area of practice, "General chiropractic" was maintained as its own category, a new "Focused practice" was created, combining "Rehabilitation chiropractic", "Pregnancy chiropractic", "Paediatric chiropractic", "Animal chiropractic", and "Diagnostic radiology"; an "Other" captured all other non-unknown responses.
7. For Community size/ geographic location, "Urban or suburban" was kept as its original category, "Geographically isolate, remote" was grouped into "Small town or rural", the "Other" category was created for responses indicating "Majority of practice is telehealth" or "Cannot identify a primary geographic location".
8. For practice format/setting, "Community – interdisciplinary medical" and "Hospital outpatient" were grouped together with "Community – interdisciplinary" into a new combined category "Community – interdisciplinary/medical", "Community – solo practice (chiropractor only)" and "Community – solo/multi dc practice" were combined into "Community – solo/multi chiropractor practice", "Community – interdisciplinary rehab" was kept as a separate category.
9. Primary payment sources were grouped into "Direct out-of-pocket" and "Third-party payer" which included direct or indirect pay through extended health insurance or public health insurance plan (provincial/territorial).
